# Immuno-inflammatory signature for predicting therapeutic response and survival after stereotactic radiosurgery in NSCLC patients with brain metastases: a retrospective cohort study

**DOI:** 10.3389/fimmu.2025.1739812

**Published:** 2025-12-18

**Authors:** Huili Zhao, Shenao Zhang, Yinjiao Wang, Haiyan Zhang, Peng Du, Aihong Cao

**Affiliations:** 1 Department of Radiology, The Second Affiliated Hospital of Xuzhou Medical University, Xuzhou, China; 2 Department of Radiology, Xinyi People’s Hospital, Xuzhou, China

**Keywords:** brain metastases, immuno-oncology, inflammatory biomarkers, neutrophil-to-lymphocyte ratio, nomogram model, non-small cell lung cancer, overall survival, stereotactic radiosurgery

## Abstract

**Purpose:**

This study aimed to delineate critical factors, particularly immune-inflammatory biomarkers, that predict therapeutic response and overall survival (OS) in non-small cell lung cancer (NSCLC) patients with brain metastases (BM) undergoing stereotactic radiosurgery (SRS), and to develop novel decision-tree and nomogram models for prognostication.

**Patients and methods:**

In this retrospective study, we analyzed data from 464 NSCLC patients with BM treated with SRS between February 2016 and November 2022. The cohort was randomly split into training and validation sets (7:3 ratio). A C5.0 algorithm was employed to build a decision tree model for treatment response. Prognostic factors for OS were identified via univariate and multivariate Cox regression, and subsequently used to construct graphical and online nomograms. Model performance was assessed with calibration curves and the C-index.

**Results:**

The median OS for the entire cohort was 15.8 months (95% confidence interval [CI]: 14.6 to 17.0 months). The decision tree model for treatment response identified NLR as a key predictor, alongside volume of brain metastases, Score Index for Radiosurgery (SIR), edema index (EI), and maximum diameter. Multivariate Cox analysis identified age, volume of brain metastases, EI, and SIR as independent prognostic factors for OS. Graphical and dynamic nomograms were developed based on these factors (available at: https://helloshinyweb.shinyapps.io/brain_metastasis_from_NSCLC/). The calibration curves demonstrated good consistency between predicted and actual survival, and the C-index indicated a moderate discriminative ability.

**Conclusions:**

We identified that immune-inflammatory profiles and radiological-clinical factors are significant predictors for treatment response and OS in NSCLC patients with BM undergoing SRS. The developed decision tree and nomogram models, which incorporate immune-inflammatory profiles, provide user-friendly tools to assist clinicians in optimizing personalized management for this patient population.

## Introduction

Lung cancer remains the predominant contributor to global cancer mortality (1), with non-small cell lung cancer (NSCLC) representing roughly 85% of all diagnosed cases (2). Clinical data reveal that 57% of NSCLC patients demonstrate multiple metastatic lesions at initial diagnosis, while 20% present with brain metastases (BM) (3). Moreover, epidemiological studies indicate approximately 40% of NSCLC patients will eventually develop BM throughout their disease progression (4). The neurological sequelae of BM, including significant cognitive impairment and diminished quality of life, substantially compromise survival outcomes (5), establishing BM as a critical determinant of therapeutic failure in NSCLC management.

The progression and treatment response of BM are not solely determined by tumor cell-intrinsic factors but are profoundly influenced by the host’s immune system and systemic inflammatory status. The tumor immune microenvironment (TIME) of brain metastases, characterized by infiltrating immune cells and cytokine networks, plays a critical role in disease progression and therapeutic outcomes (6). Furthermore, systemic inflammatory markers, such as the neutrophil-to-lymphocyte ratio (NLR), serve as accessible proxies of the host’s immune-inflammatory balance and have been implicated in predicting survival and treatment efficacy across various cancers, including NSCLC (7).

Contemporary management of BM in NSCLC prioritizes neurological symptom management and quality of life preservation through multimodal interventions. Therapeutic approaches encompass localized modalities (surgical resection, whole brain radiotherapy [WBRT], stereotactic radiosurgery [SRS]) and systemic regimens (chemotherapeutic agents, molecularly targeted agents, immunotherapeutic strategies) (6). Emerging evidence favors SRS implementation in patients with oligometastatic intracranial disease (≤4 lesions) due to its superior efficacy profile, abbreviated treatment schedules, and minimized neurotoxicity compared to conventional radiotherapy (7, 8). Level 1 evidence establishes SRS monotherapy as the standard of care for this patient population (9).

Precision medicine has driven the development of prognostic stratification tools that serve as critical adjuncts for optimizing oncological care. Decision tree models - supervised machine learning algorithms - generate classification frameworks for outcome prediction through recursive data partitioning. Concurrently, nomograms statistically synthesize multidimensional clinical parameters (demographic, histopathological, imaging, and treatment variables) to compute personalized survival estimates (10, 11). The digital implementation of these calculators through web-based platforms significantly enhances their clinical utility by enabling real-time prognostic assessment.

This investigation conducted a retrospective cohort analysis of NSCLC patients with BM receiving SRS to delineate determinants of therapeutic response and survival outcomes, with a specific focus on the role of immune-inflammatory biomarkers. Through this analytical framework, we constructed and validated three prognostic instruments: machine learning-derived decision trees, clinically accessible nomograms, and the first reported web-deployable calculator enabling real-time prognostic estimation in this patient population.

## Materials and methods

### Data source and collection

Data from NSCLC patients with BM who underwent SRS therapy at The Second Affiliated Hospital of Xuzhou Medical University between February 2016 and November 2022 were reviewed. This study was approved by the Institutional Review Board of the Second Affiliated Hospital of Xuzhou Medical University and conducted in accordance with relevant guidelines/regulations and the Declaration of Helsinki. This study is a retrospective study, and the used data collected as part of the participants’ routine care. Written informed consent for participation was waived for this study in accordance with the national legislation and the institutional requirements.

### Inclusion and exclusion criteria

Inclusion criteria: (1) Pathologically confirmed NSCLC; (2) No more than four BM; (3) Exclusive SRS therapy; (4) KPS ≥ 70; (5) Complete pre-therapy and follow-up MRI, including T1 weighted imaging (T1WI), T2 weighted imaging (T2WI), diffusion weighted imaging (DWI), and contrast enhanced T1 weighted imaging (CE-T1WI); (6) Complete records of clinical, pathological, and treatment information. Exclusion criteria: (1) Pathologically confirmed combined with another primary cancer; (2) Prior craniocerebral surgery or WBRT before SRS therapy; (3) Concurrent chemotherapy or immune checkpoint inhibitor therapy within one month before or after SRS; (4) Cystic or cranial metastases.

### Patients information collection

Patients information included: (1) Clinical information: sex, age, KPS (before SRS therapy), Score Index for Radiosurgery (SIR), neutrophil-to-lymphocyte ratio (NLR) (a key systemic inflammatory marker measured from peripheral blood before surgery), CEA (normal or abnormal), CA125 (normal or abnormal), Cyfra-211 (normal or abnormal), pathological type [squamous cell carcinoma (SCC), adenocarcinoma, adenosquamous carcinoma (AC), large cell carcinoma (LCC), or others], Ki-67 (≤ 30% or > 30%), epidermal growth factor receptor (EGFR) (mutation or non-mutation), anaplastic lymphoma kinase (ALK) (mutation or non-mutation), mediastinal lymph node metastases before surgery (yes or no), postoperative chemotherapy (yes or no), primary tumor control (yes or no), interval between NSCLC confirmation and presence of BM (≤ 3 months or > 3 months), neurological symptoms (yes or no), number of BM (1 or 2-4), extracranial metastasis (yes or no); (2) Radiological information of BM: location, volume of brain metastases (measured on CE-T1WI rather than the total tumor burden), maximum diameter, edema index (EI), T1WI signal intensity, T2WI signal intensity, DWI signal intensity, and enhanced pattern.

For patients with multiple brain metastases, the sum of the volumes of all measurable metastases (up to a maximum of 4, per inclusion criteria) was calculated and used for analysis. Volumetric measurements were performed manually by contouring the border of each metastasis on every slice of the axial CE-T1WI sequences using the PACS (Picture Archiving and Communication System) software, which automatically calculates the volume based on the contoured area and slice thickness.

### Treatment and efficacy evaluation

Patients underwent SRS therapy using the Leksell Gamma Knife® Perfexion™ (Elekta, Norcross, GA, USA). All patients received the prescribed dose in a single fraction.

The prescription dose was individualized based on the tumor volume and location, following our institutional protocol. The median margin dose was 18 Gy (range 16–20 Gy), and the specific distribution was as follows: 92 patients (19.8%) received 16 Gy, 301 patients (64.9%) received 18 Gy, and 71 patients (15.3%) received 20 Gy. Key dosimetric parameters, including the prescription isodose line (median 50%, range 50%-70%) and the maximum dose, were recorded. A preliminary analysis found no significant correlation between these dosimetric parameters and OS or treatment response in our cohort, likely because the dose variation was protocol-driven and relatively narrow. Therefore, to maintain model parsimony and clinical applicability, they were not included as candidate variables in the final predictive models.

Pre-therapy MRI served as the baseline for assessing treatment response, with follow-up MRI performed every 3 months post-SRS. All of the MRI examinations were conducted using a 1.5T MRI system (uMR 588 1.5T; United Imaging Healthcare, Shanghai, China) with an 8-channel phased-array head coil. MRI sequences included T1WI, T2WI, DWI, and CE-T1WI.

Treatment responses were evaluated based on the pre-therapy MRI and the MRI approximately 3 months post-SRS, using the Response Assessment in Neuro-Oncology Brain Metastases (RANO-BM) criteria (12).Patients were categorized into complete response (CR), partial response (PR), stable disease (SD), and progressive disease (PD). OS was defined as the time from BM diagnosis to the last follow-up or to death, with a follow-up deadline of July 30, 2024.

### Statistical analysis

Continuous variables such as age, volume of brain metastases, maximum diameter, and EI were dichotomized to facilitate their integration into the decision-tree model. The optimal cut-off points for dichotomizing continuous variables were determined by maximizing the separation of survival curves using the maximally selected log-rank statistics (via the ‘surv_cutpoint’ function from the ‘survminer’ package in R). This approach was chosen over median split or ROC analysis because it is specifically suited for identifying prognostically relevant thresholds in time-to-event data. We acknowledge the exploratory nature of this method. Therefore, a sensitivity analysis was conducted using the original continuous variables to confirm the robustness of the prognostic factors identified in the Cox model.

**Figure 1:**
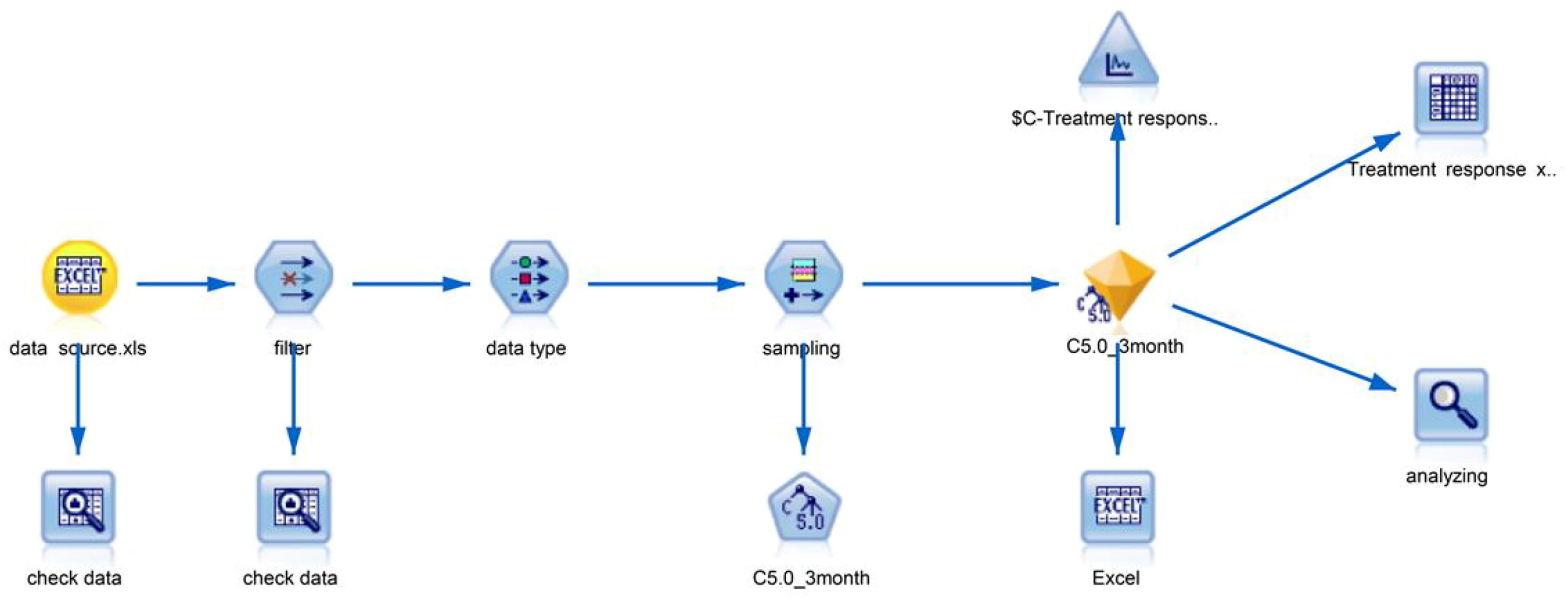
The analysis stream of decision tree development and validation, including processes of data input, data filtering, data type determination, model training, and model testing.

Patients were randomly divided into the training and validation sets at a ratio of 7:3. Using the training sample, a decision tree model was developed, by C5.0 algorithm. The depth of the decision tree was limited within 5 layers. The relative importance of the predictive factors was calculated by the C5.0 algorithm using the information gain metric. The analysis stream of decision tree development and validation is available in **Figure 1**, including processes of data input, data filtering, data type determination, model training, and model testing. In the process of model testing, a scatter plot was generated to evaluate the prediction accuracy of the decision tree. After that, univariable Cox analysis was proceeded for all predictive factors, to filter the potentially important prognosis factors, which were contained in the multivariable Cox model.

Graphical and online nomograms were developed using significant factors from the multivariate analysis. The graphical nomogram was constructed based on the final multivariate Cox model. The points assigned to each variable level in the nomogram were derived by scaling the corresponding regression coefficients (β), typically by the largest coefficient among all predictors (13). These points are then summed to obtain a ‘Total Points’ score, which is projected onto the bottom scales to estimate survival probabilities and median survival time.

The online dynamic nomogram is hosted on a secure server, and we commit to maintaining its availability and functionality for at least two years from the date of publication. The code and underlying data schema will be archived to ensure future reproducibility.

Calibration curve and C-index were used to assess model accuracy and discrimination.

All statistical analyses were performed using R version 4.1.3, with a significance level of 0.05.

The key R packages used for analysis included: rms (version 6.7-0) for nomogram construction and calibration curves, survival (version 3.5-7) for Cox regression, time ROC (version 0.4) for time-dependent ROC analysis, and dplyr (version 1.1.4) for data handling.

## Results

### Baseline characteristics

A total of 464 patients met the inclusion criteria, with 325 and 139 patients in the training and validation sets, respectively. Baseline characteristics are summarized in **Table 1**. The median OS for the entire cohort was 15.8 months (95% confidence interval [CI]: 14.6 to 17.0 months). The Log-rank test showed that the optimum cut-off points for age, EI, maximum diameter and volume of brain metastases were 48, 4.11, 3.15 cm and 3.10 cm^3^, respectively (**Supplementary Figure S1**). The optimal cut-off for age (48 years) was derived statistically to maximize survival curve separation, which is lower than conventional clinical thresholds. Given that the statistically derived cut-off for age was considerably lower than the median age and conventional clinical thresholds, we performed a sensitivity analysis using a clinically relevant cut-off of 60 years. When the multivariate Cox model was refitted with age dichotomized at 60 years, it remained an independent prognostic factor (HR = 1.82, 95% CI: 1.25-2.65, *P* = 0.002), and the model’s C-index was similar (0.66). This confirms that older age is a robust predictor of poorer OS, though the magnitude of the effect is more clinically plausible when using a conventional age cut-off.

**Table 1:** Baseline characteristics of the enrolled patients.

Variables	Whole cohort (n=464)	Training sample (n=325)	Validation sample (n=139)	*P* value
Clinical Characteristics				
Sex, n (%)				0.842
Female	240 (51.7%)	167 (51.4%)	73 (52.5%)	
Male	224 (48.3%)	158 (48.6%)	66 (47.5%)	
Age (years)	62 [55, 69]	62 [55, 69]	63 [56, 70]	0.415
KPS, n (%)				0.874
70-80	96 (20.7%)	68 (20.9%)	28 (20.1%)	
90-100	368 (79.3%)	257 (79.1%)	111 (79.9%)	
SIR, n (%)				0.589
I	64 (13.8%)	48 (14.8%)	16 (11.5%)	
II	320 (69.0%)	220 (67.7%)	100 (71.9%)	
III	80 (17.2%)	57 (17.5%)	23 (16.6%)	
NLR	2.9 [2.0, 4.2]	3.0 [2.1, 4.3]	2.8 [1.9, 4.1]	0.187
Pathological type, n (%)				0.945
Adenocarcinoma	352 (75.9%)	247 (76.0%)	105 (75.5%)	
SCC	72 (15.5%)	51 (15.7%)	21 (15.1%)	
Others	40 (8.6%)	27 (8.3%)	13 (9.4%)	
EGFR, n (%)				0.184
Mutation	255 (55.0%)	185 (56.9%)	70 (50.4%)	
Wild-type	209 (45.0%)	140 (43.1%)	69 (49.6%)	
Extracranial metastasis, n (%)				0.065
Yes	200 (43.1%)	131 (40.3%)	69 (49.6%)	
No	264 (56.9%)	194 (59.7%)	70 (50.4%)	
Radiological Characteristics				
Number of BM, n (%)				0.788
1	245 (52.8%)	173 (53.2%)	72 (51.8%)	
2-4	219 (47.2%)	152 (46.8%)	67 (48.2%)	
Volume of BM (cm^3^)	2.00 [0.80, 4.80]	1.95 [0.78, 4.75]	2.10 [0.83, 4.90]	0.321
Maximum diameter (cm)	2.5 [1.7, 3.5]	2.5 [1.7, 3.5]	2.6 [1.7, 3.6]	0.456
Edema Index (EI)	2.1 [1.4, 3.6]	2.0 [1.3, 3.5]	2.2 [1.5, 3.8]	0.274
Location, n (%)				0.887
Frontal lobe	88 (18.9%)	61 (18.8%)	27 (19.4%)	
Occipital lobe	80 (17.3%)	50 (15.4%)	30 (21.6%)	
Temporal lobe	71 (15.3%)	51 (15.7%)	20 (14.4%)	
Parietal lobe	63 (13.6%)	47 (14.5%)	16 (11.5%)	
Cerebellum	109 (23.5%)	76 (23.4%)	33 (23.7%)	
Brainstem	32 (6.9%)	24 (7.4%)	8 (5.8%)	
Others†	21 (4.5%)	16 (4.9%)	5 (3.6%)	

Data are presented as n (%) for categorical variables and Median [IQR] for continuous variables.

KPS, Karnofsky Performance Status; SIR, Score Index for Radiosurgery; NLR, neutrophil-to-lymphocyte ratio; SCC, squamous cell carcinoma; BM, brain metastases; IQR, interquartile range.

†The ‘Others’ category for location included metastases located in the basal ganglia, thalamus, corpus callosum, and ventricular system.

*P values for continuous variables were calculated using the Mann-Whitney U test. P values for categorical variables were calculated using the Chi-square test or Fisher’s exact test. Fisher’s exact test was used when more than 20% of the expected cell counts were less than 5.

### Decision tree model development and validation

The therapeutic response to SRS, evaluated at 3 months using RANO-BM criteria, is summarized for both cohorts in **Supplementary Table S1**. In the entire cohort, the objective response rate (ORR, comprising CR and PR) was 68.1% (316/464), while the disease control rate (DCR, comprising CR, PR, and SD) was 89.4% (415/464). The distribution of responses was well-balanced between the training and validation sets (ORR: 67.7% vs. 69.1%; DCR: 89.2% vs. 90.0%).

**Figure 2:**
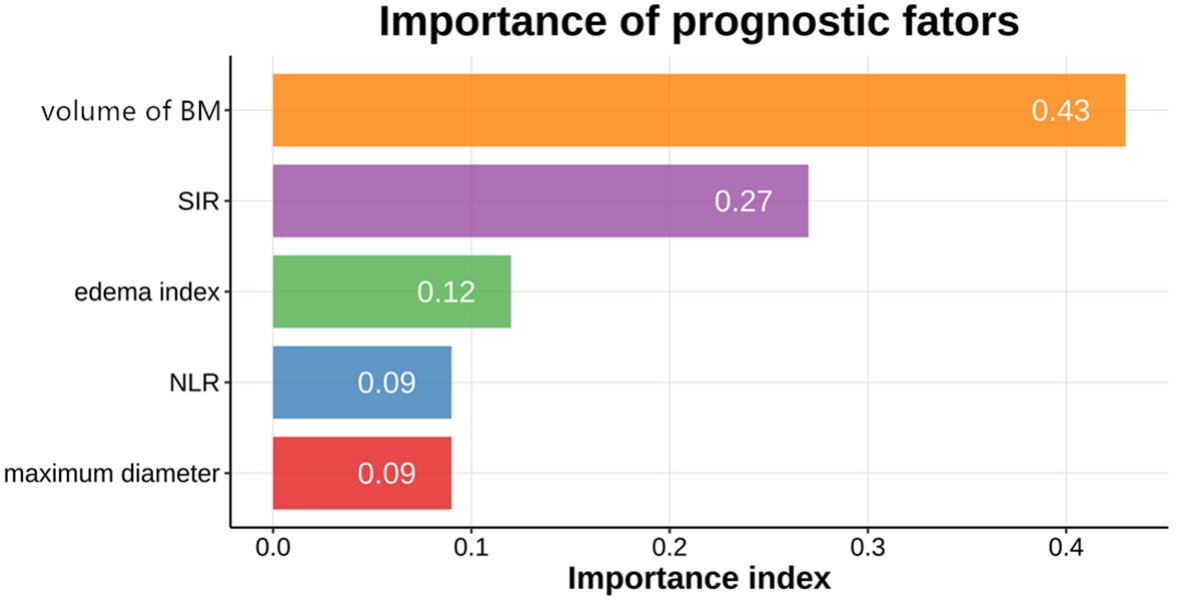
The order of importance of the predictors for the decision tree model, as determined by the C5.0 algorithm using the information gain metric. BM, brain metastases; SIR, Score Index for Radiosurgery; NLR, neutrophil-to-lymphocyte ratio.

**Figure 3:**
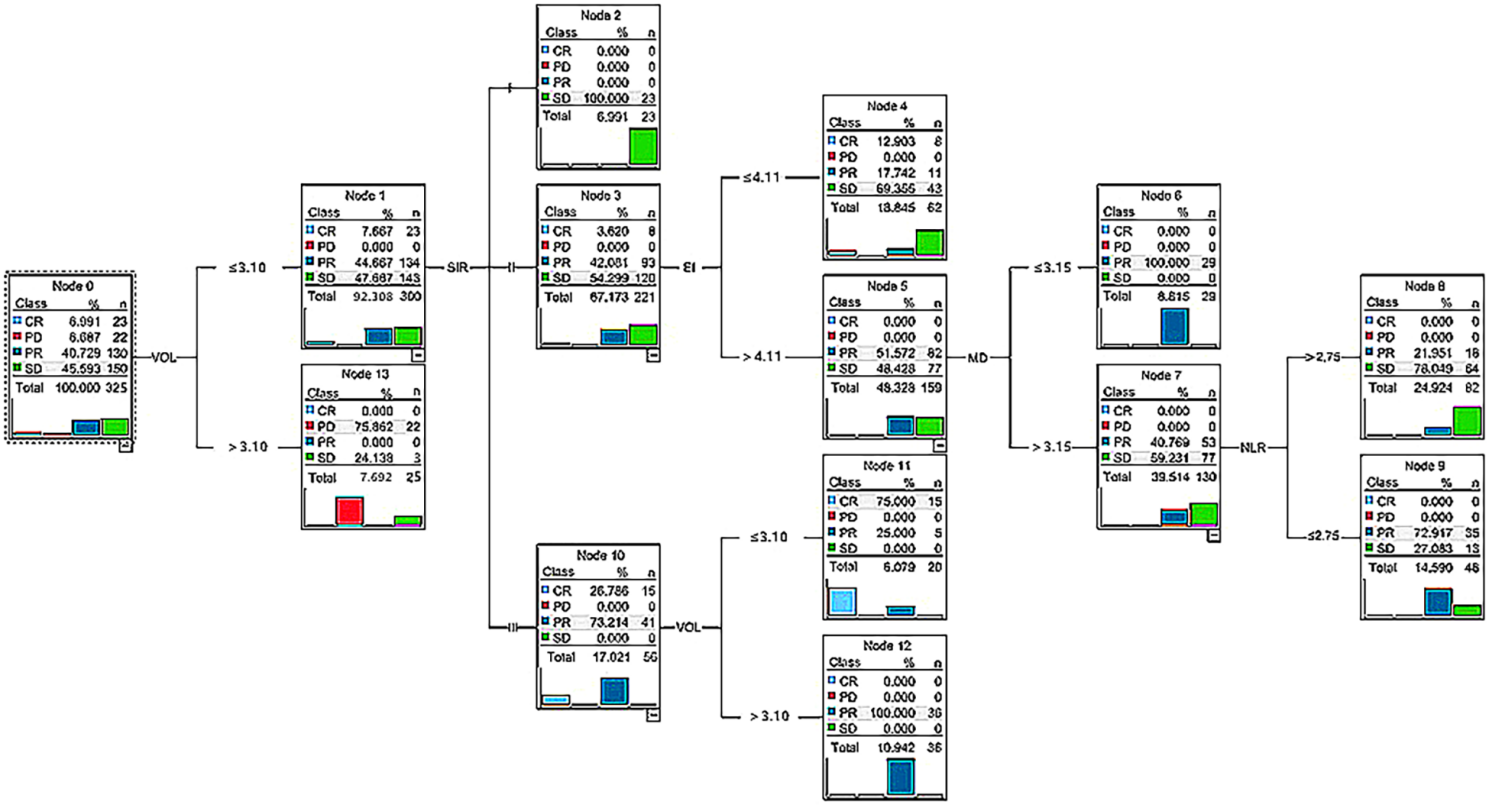
The decision tree model established using the C5.0 algorithm. Volume of brain metastases, SIR, edema index, maximum diameter, and NLR were of primary importance for treatment response prediction. Each node in the tree displays the number of patients (n) and the distribution of treatment responses (CR/PR/SD/PD) for clarity. SIR, Score Index for Radiosurgery; NLR, neutrophil-to-lymphocyte ratio; MD, Maximum Diameter.

The importance of predictive factors for treatment response is shown in **Figure 2**. Volume of brain metastases, SIR, EI, maximum diameter, and NLR (a systemic immune-inflammatory marker) were identified as the most influential predictors based on their relative importance. The decision tree model for treatment response is shown in **Figure 3**.

To illustrate the clinical utility and interpretability of the decision tree model, we present two representative patient pathways based on its specific splitting criteria: Case Example 1 (Predicted Favorable Responder): Consider a patient with a brain metastasis volume of 2.5 cm^3^, classified as SIR grade II, and presenting with mild peritumoral edema (EI = 2.5). This patient would be stratified directly down the leftmost branch of the tree to a terminal node associated with a high probability of CR or PR. This prediction identifies a patient who is an excellent candidate for SRS monotherapy with a high anticipated likelihood of local control. Case Example 2 (Predicted Poor Responder): Now, consider a different patient also with a metastasis volume of 2.8 cm^3^ and SIR grade II, but exhibiting significant peritumoral edema (EI = 5.5). The tree would then further evaluate the lesion’s maximum diameter. If the diameter measures 3.5 cm, and the pre-treatment NLR is elevated at 3.8, the patient would be guided to a terminal node dominated by SD or PD. This stratification alerts the clinician to a substantially higher risk of suboptimal response to SRS. This information could prompt considerations for more intensive post-treatment monitoring, earlier follow-up imaging, or the discussion of complementary or alternative treatment strategies at the outset.

**Figure 4:**
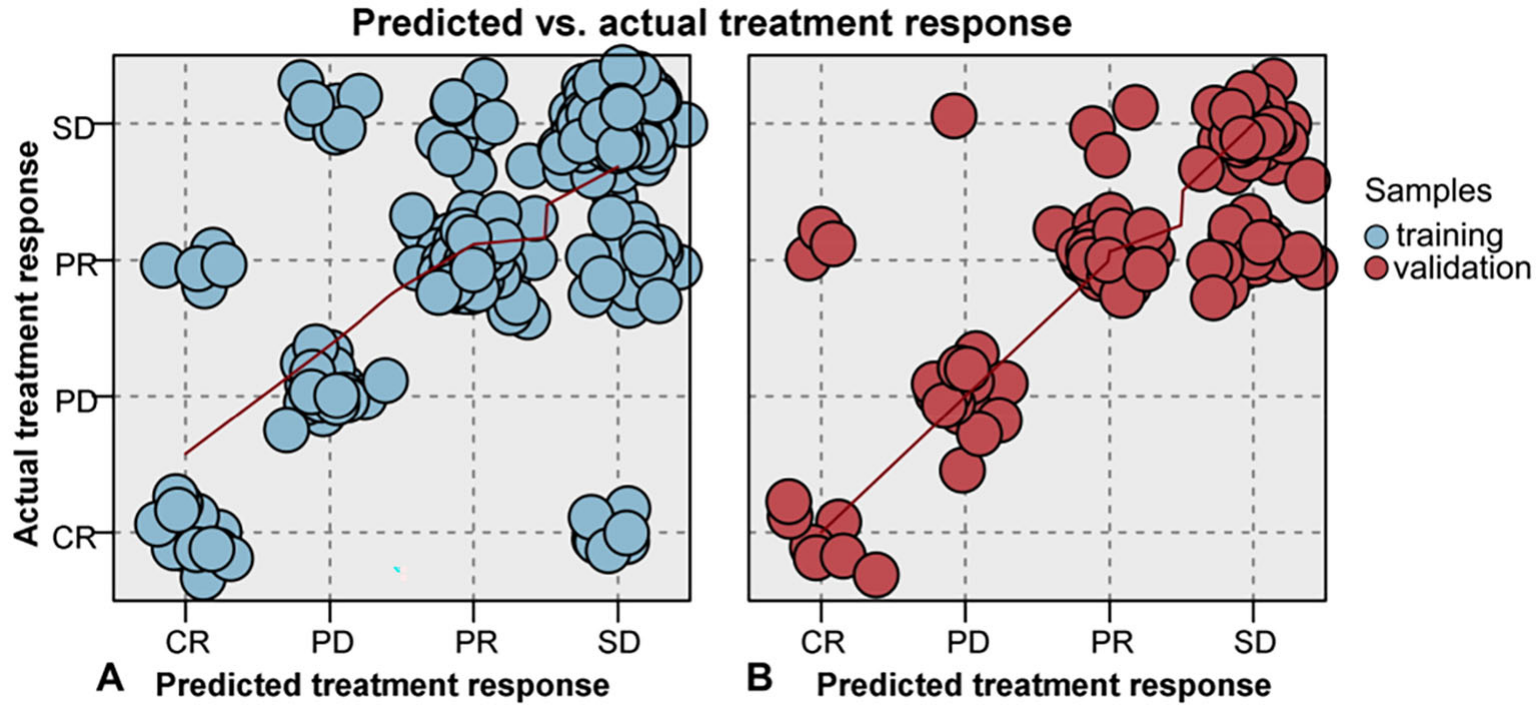
The scatter plot for evaluating the consistency between predicted and actual treatment response, both for training **(A)** and validation **(B)** samples. Most of the scatters are located on the diamond line, and the fitted line is closed to the diamond line, demonstrating excellent consistency.

**Figure 4** shows the scatter plot for assessing the consistency between predictive and actual treatment response, for training and validation samples. Preferable consistency between the predictive and actual survival status was presented in the 2 samples. Most of the scatters are located on the diamond line, and the fitted line is closed to the diamond line, demonstrating excellent consistency. The accuracy rates were 81.16% and 80.74% for the training and validation sets, respectively. To further evaluate the model’s performance across all response categories, the macro-averaged F1-score was calculated, yielding values of 0.79 and 0.78 for the training and validation sets, respectively. This indicates that the model maintains a good and consistent balance between precision and recall for each class of therapeutic response.

To evaluate the model’s generalizability, we assessed its discriminative ability (C-index) in key clinical subgroups within the validation set. The model demonstrated consistent performance across most subgroups, including patients with and without EGFR mutations, and with and without extracranial metastases (see **Supplementary Table S2**).

### Univariable and multivariable Cox analyses

Univariate analysis identified maximum diameter (HR = 2.331, 95%CI: 1.710-3.178, *P* < 0.001), EI (HR = 4.681, 95%CI: 3.294-6.653, *P* < 0.001), age (HR = 1.726, 95%CI: 1.129-2.640, *P* = 0.012), volume of brain metastases (HR = 6.332, 95%CI: 3.856-10.398, *P* < 0.001), enhanced pattern (HR = 1.213, 95%CI: 1.047-1.406, *P* = 0.010) and SIR (grade I vs. grade II: HR = 0.454, 95%CI: 0.288-0.717, *P* < 0.001; grade I vs. grade III: HR = 0.206, 95%CI: 0.119-0.357, *P* < 0.001) were identified to be significantly relevant to the OS (*P* < 0.05).

**Figure 5:**
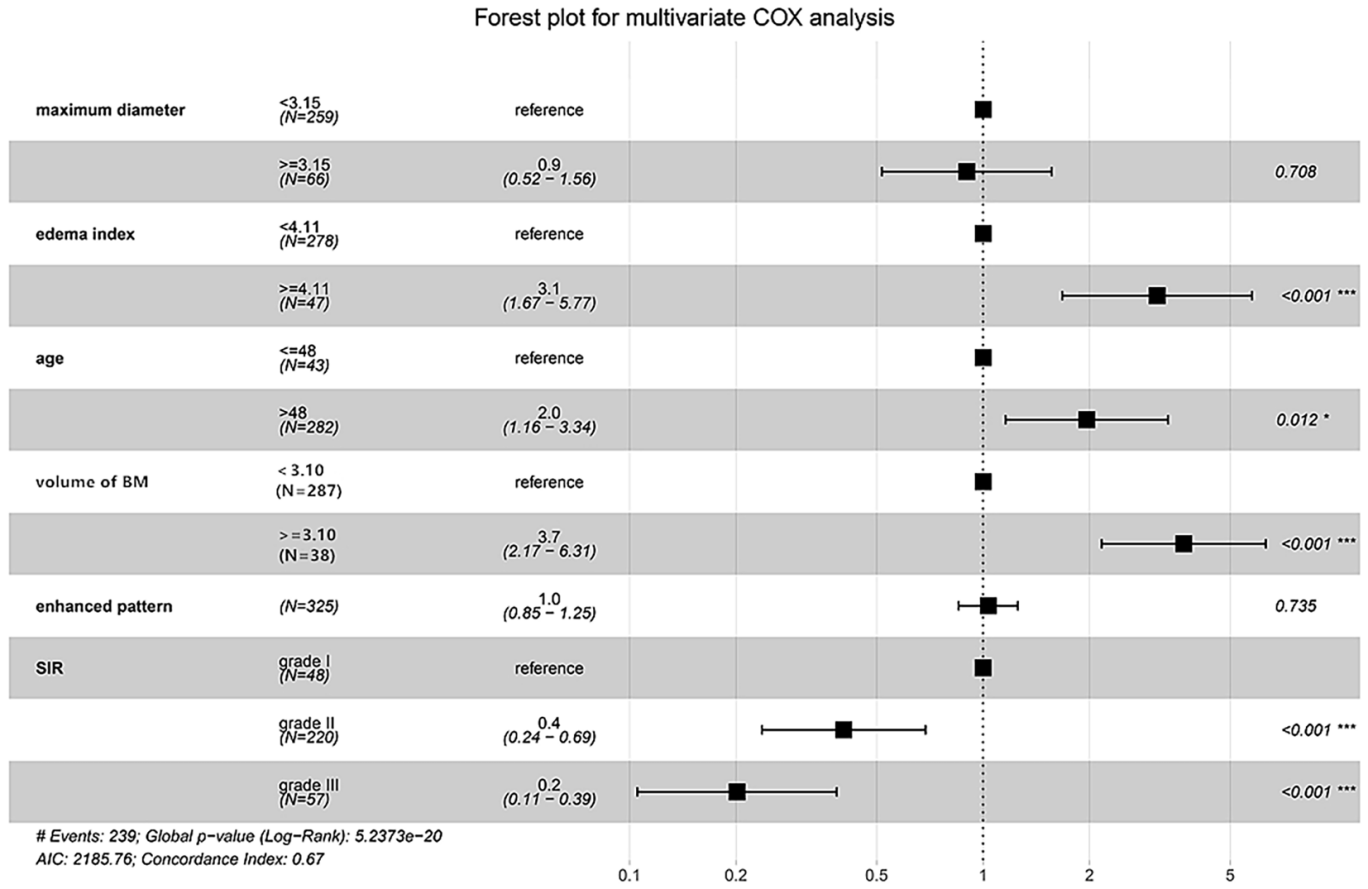
Forest plot for the multivariable Cox analysis of overall survival. Age, volume of brain metastases, edema index, and SIR were identified as the significant prognostic factors. BM, brain metastases; SIR, Score Index for Radiosurgery.

**Figure 6:**
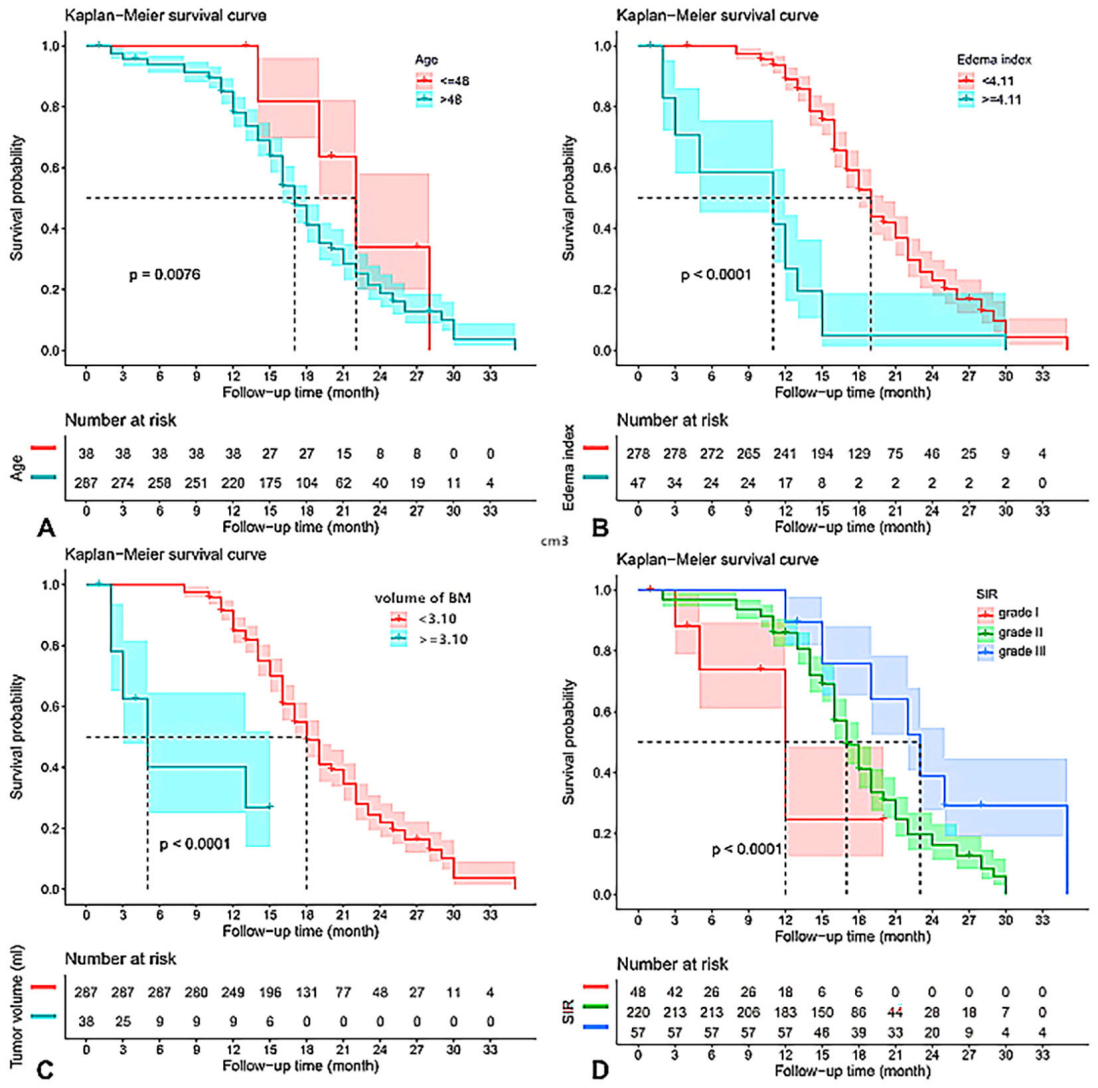
Kaplan-Meier survival curves for age **(A)**, edema index **(B)**, volume of brain metastases **(C)**, and SIR **(D)** which were significantly associated with overall survival in multivariable analysis. BM, brain metastases; SIR, Score Index for Radiosurgery.

The results of the univariate and multivariate Cox regression analyses for OS are summarized in **Table 2**. In univariate analysis, maximum diameter, EI, age, volume of brain metastases, enhanced pattern, and SIR were significantly associated with OS. These significant variables were subsequently incorporated into the multivariate Cox model. After multivariable adjustment, age, volume of brain metastases, EI, and SIR were ultimately identified as independent prognostic factors for OS (**Figure 5**). Notably, maximum diameter, which was significant in univariate analysis, was not retained in the final multivariable model, likely due to its collinearity with the volume of brain metastasis and its incorporation within the SIR score. A sensitivity analysis was conducted using the original continuous variables for age, EI, and volume in the multivariate Cox model. The results corroborated the primary findings, with all factors retaining their independent prognostic significance (see **Supplementary Table S3**). The Kaplan-Meier survival curves for these significant factors are shown in **Figure 6**.

**Table 2:** Univariate and multivariate Cox regression analyses of factors associated with overall survival.

Variable	Univariate analysis		Multivariate analysis	
	HR (95% CI)	*P* value	HR (95% CI)	*P* value
Age (>48 vs. ≤48 years)	1.73 (1.13 - 2.64)	0.012	3.11 (1.67 - 5.77)	<0.001
Volume of BM (≥3.10 vs. <3.10 cm^3^)	6.33 (3.86 - 10.40)	<0.001	3.70 (2.17 - 6.31)	<0.001
Edema Index (≥4.11 vs. <4.11)	4.68 (3.29 - 6.65)	<0.001	1.97 (1.16 - 3.34)	0.012
Maximum diameter (≥3.15 vs. <3.15 cm)	2.33 (1.71 - 3.18)	<0.001	-	-
SIR				
Grade I (Reference)	1.00		1.00	
Grade II vs. I	0.45 (0.29 - 0.72)	<0.001	0.40 (0.24 - 0.69)	0.001
Grade III vs. I	0.21 (0.12 - 0.36)	<0.001	0.20 (0.11 - 0.39)	<0.001
Enhanced pattern	1.21 (1.05 - 1.41)	0.010	-	-

Only variables with a P value < 0.05 in the univariate analysis were included in the multivariate Cox proportional hazards model.

HR, Hazard Ratio; CI, Confidence Interval; BM, Brain Metastases; SIR, Score Index for Radiosurgery.

### Establishment and validation of the nomogram model

**Figure 7:**
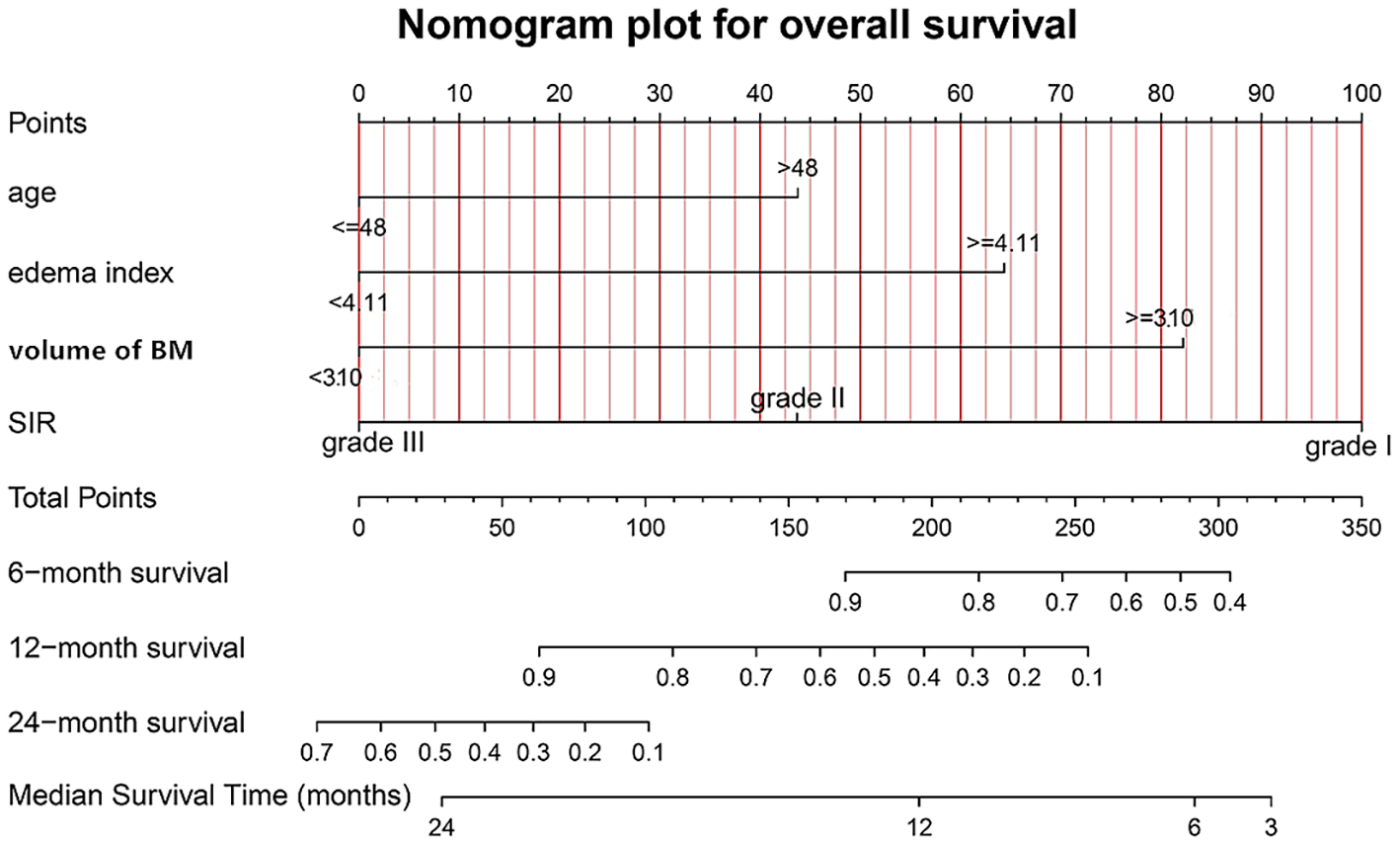
The graphical nomogram model involving the four independent prognostic indicators (age, volume of brain metastases, edema index, and SIR) for overall survival, to predict the patients’ 6-, 12-, and 24-month survival and median survival time. BM, brain metastases; SIR, Score Index for Radiosurgery.

**Figure 7** presents the graphical nomogram for predicting 6-, 12-, and 24-month survival and median survival time based on the four independent prognostic factors.

The calibration curves at 6, 12, and 24 months, for training (A-C) and validation (D-F) samples, are shown in **Supplementary Figure S2**. The calibration curves demonstrated good agreement between predicted and observed survival probabilities, with the fitted lines closely approximating the ideal 45-degree line, indicating satisfactory model calibration. The C-indexes were 0.67 (95%CI: 0.59-0.75) and 0.66 (95%CI: 0.53-0.80) for the training and validation samples, respectively. This indicates a moderate yet consistent discriminative ability of the nomogram in both cohorts. The relatively wide confidence interval in the validation set suggests some instability in model performance on external data, which highlights an area for future refinement and validation in larger cohorts.

To benchmark the performance of our nomogram, we compared its C-index in the validation set against that of the Disease-Specific Graded Prognostic Assessment for lung cancer using molecular markers (Lung-molGPA). The C-index of our nomogram was 0.66 in the validation set, which represents a moderate level of predictive accuracy. Although the C-index of our nomogram (0.66) was comparable to or slightly higher than that of the Lung-molGPA score (0.59) in our cohort, this finding primarily establishes the clinical feasibility and utility of integrating radiological features and systemic inflammation markers for prognosis in this patient population. We acknowledge that there is room for improvement in the model’s discriminative power. The moderate C-index underscores the complex and multifactorial nature of overall survival, which is influenced by unmeasured variables such as subsequent lines of therapy and detailed molecular profiles. Nevertheless, this model provides a solid foundation for future research. We envision that incorporating dynamic changes in inflammatory markers, more comprehensive genomic data, and features of the tumor immune microenvironment could further enhance the model’s performance.

### Online dynamic nomogram model establishment

**Figure 8:**
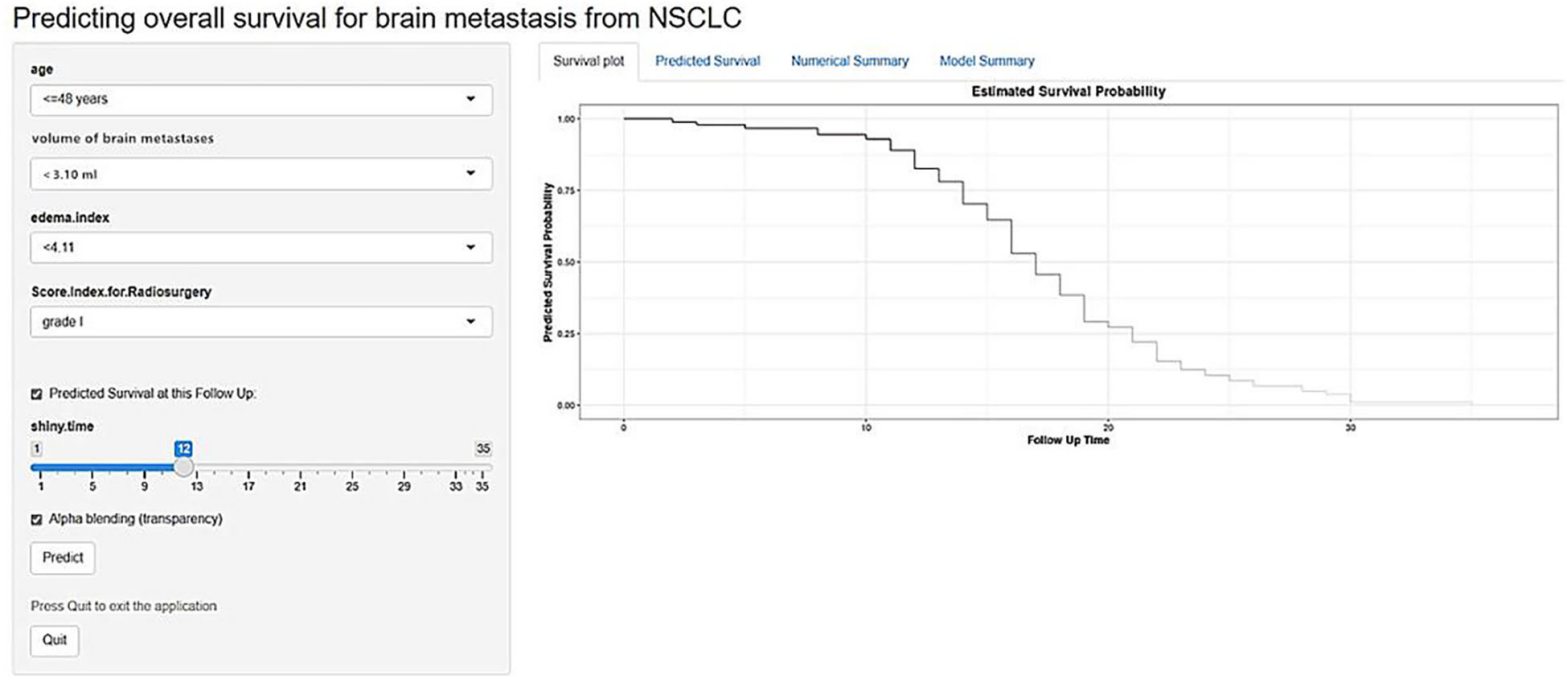
The screenshot of the online prediction tool for overall survival.

Based on the predictive factors identified by multivariable Cox model and the graphical nomogram, an online dynamic nomogram model for OS was established. The online dynamic nomogram is currently accessible as a demonstration version at: https://helloshinyweb.shinyapps.io/brain_metastasis_from_NSCLC/. Upon acceptance of the manuscript, the final tool will be migrated to a stable institutional server with a long-term commitment to maintenance. A screenshot of the online tool is shown in **Figure 8**.

## Discussion

Lung cancer is the leading cause of cancer related deaths worldwide, with NSCLC being the most common sub-type (14–16). This immuno-oncology study retrospectively analyzed clinicopathological, radiological, and immune-inflammatory data of 464 NSCLC patients with BM treated with SRS to identify factors influencing treatment response and OS, and to guide individualized treatment planning. Our findings underscore the significant prognostic value of integrating systemic immune-inflammatory status and local radiological features in predicting outcomes for these patients.

Adenocarcinoma is more likely to develop BM due to its biological behavior and ability to metastasize via the bloodstream (17–19). While this study did not find a significant correlation between NSCLC pathological types and prognosis, possibly due to the limited sample size, it is noteworthy that different histological subtypes can exhibit distinct immune microenvironments, which may influence metastatic patterns and treatment responses (20–22). Future studies with larger cohorts are needed to explore this relationship further from an immunological perspective.

The presence or absence of EGFR and ALK mutations was not found to be an independent prognostic factor for OS in this study. The most plausible explanation for this finding is the lack of detailed data on systemic therapies administered after SRS, which is a key determinant of long-term survival and a major limitation of our retrospective design. Other potential reasons include the modulatory effect of the tumor immune microenvironment, insufficient follow-up time to observe long-term interactions, and the potential influence of other genomic alterations or individual differences in anti-tumor immunity (23–26).

The treatment response was evaluated at approximately 3 months post-SRS, a time point commonly used in clinical trials and practice to allow for the full effect of radiation to manifest while avoiding the confounding effects of later-occurring radionecrosis. However, we recognize that early pseudoprogression, typically occurring within the first 1–3 months, can sometimes mimic true progression. While the RANO-BM criteria used in this study help mitigate this issue by incorporating clinical and steroid-use status, our retrospective design cannot entirely rule out misclassification in a small number of cases. This is a known limitation of SRS response assessment. Future studies with more frequent, serial MRI and advanced imaging would be valuable to dynamically capture the treatment response trajectory.

This study identified a combination of radiological parameters and a systemic immune-inflammatory marker that predict outcomes following SRS. Specifically, volume of brain metastases, SIR, EI, maximum diameter, and NLR were key predictors of the initial treatment response. For long-term survival, age, SIR, volume of brain metastases, and EI were independent prognostic factors.

In our cohort, SRS demonstrated high efficacy, with a disease control rate of 89.4% and an objective response rate of 68.1% at 3 months (**Supplementary Table S1**). This aligns with the established efficacy profile of SRS for managing brain metastases. Beyond reporting these overall efficacy rates, the primary aim of our study was to develop a decision-tree model for predicting individual patient response prior to treatment, using readily available clinical and radiological factors. The high accuracy of our model underscores its potential utility in clinical decision-making.

SIR, which incorporates tumor volume as a key component, was a robust predictor. Larger tumor volume not only presents a physical challenge for SRS but is also often associated with an immunosuppressive microenvironment, hindering effective anti-tumor immunity and contributing to poorer outcomes (27–31). Our finding that maximum diameter is a key factor in a specific patient subgroup further highlights the intricate relationship between tumor geometry, the local environment, and likely, the immune context.

EI, a measure of peritumoral edema, emerged as a significant independent prognostic factor. We propose that EI serves as a radiological surrogate for the local inflammatory and immune response within the brain parenchyma. Peritumoral edema is a manifestation of vasogenic edema, driven by inflammatory mediators and cytokine release from the tumor and surrounding stromal cells (6, 32–35). An elevated EI (≥4.11 for OS) likely reflects a more pronounced pro-inflammatory and potentially immunosuppressive peri-tumoral niche, which may facilitate tumor progression and resistance to therapy (36–38). Therefore, EI should be considered a significant parameter in the radiological and immunological evaluation of BM.

Age was confirmed as an independent prognostic factor in our model. The statistically derived cut-off of 48 years is lower than conventional clinical thresholds. This may identify a subgroup of younger patients with distinct tumor biology or resilience that confers a pronounced survival advantage. However, the robustness of age as a prognostic factor is strengthened by our sensitivity analysis, which demonstrated consistent significance using a conventional cut-off of 60 years.

The prominence of NLR in our decision tree model for treatment response underscores the critical role of systemic host immunity and its interplay with the local tumor microenvironment (39–41). A high NLR reflects a systemic state of neutrophilia and relative lymphocytopenia. Neutrophils can promote an immunosuppressive niche by secreting factors like arginase-1 and reactive oxygen species, which inhibit the cytotoxic function of T cells and NK cells, and by releasing pro-angiogenic factors like VEGF (42–45). Conversely, lymphocytes, particularly cytotoxic T cells, are essential for mediating anti-tumor immunity (46, 47).

The interplay between the systemic inflammatory state, reflected by NLR, and the local peritumoral environment, indicated by EI, is of particular interest. We postulate that these factors are not independent but rather represent interconnected components of a “systemic-local immune-inflammatory axis” in BM. A elevated NLR may signify a state of systemic immune dysregulation that fosters a permissive environment for the development of pronounced peritumoral edema at the metastatic site. Conversely, the inflammatory mediators driving vasogenic edema in the brain parenchyma could further exacerbate systemic inflammation. This vicious cycle could contribute to a more aggressive disease phenotype and poorer outcomes. Our findings, which identify both NLR and EI as key predictors, provide clinical support for this concept and underscore the importance of integrating both systemic and local inflammatory metrics for comprehensive prognostic assessment.

SRS is known to induce immunogenic cell death, releasing tumor antigens that can potentiate a systemic anti-tumor T-cell response. A high pre-treatment NLR may indicate an immune context that is less capable of mounting this effective anti-tumor immune response post-SRS, thereby explaining its association with poorer local control (48–51).

The finding that NLR was a key predictor for initial treatment response in the decision tree but not an independent prognostic factor for OS in the Cox model is noteworthy. This suggests that the systemic inflammatory state reflected by the baseline NLR may have a more direct impact on the immediate radiotherapeutic effect on the tumor microenvironment. In contrast, long-term survival is influenced by a more complex array of factors beyond initial local control, including the efficacy of subsequent systemic therapies, control of extracranial disease, and the development of new metastases.

The finding that NLR predicted initial treatment response but was not an independent prognostic factor for OS in our multivariate model is intriguing but not uncommon. This apparent discordance can be explained by the fact that long-term survival in patients with BM is influenced by a complex array of factors beyond local control, including the efficacy of subsequent systemic therapies, control of extracranial disease, and the development of new metastases. A robust initial response may not translate into a survival advantage if these other factors are unfavorable. Furthermore, the immune context reflected by baseline NLR may be more directly linked to the immediate radiotherapeutic effect on the tumor microenvironment than to long-term survival determinants. Importantly, our supplementary analysis confirmed that achieving an early radiological response (CR/PR) was, as expected, a powerful independent predictor of longer OS. This finding strongly supports the clinical value of achieving a good initial response. While we deliberately maintained a separation between our pre-treatment prognostic tools (the nomogram for OS and the decision tree for response) to enhance clinical clarity and methodological rigor, the strong association between early response and survival opens a clear path for future research to develop dynamic prediction models that integrate post-treatment response data.

The C-index of our nomogram demonstrated moderate predictive accuracy in both the training and validation cohorts. This establishes a solid foundation for future research and indicates a clinically useful level of discrimination, particularly when compared to existing tools like the Lung-molGPA. This model provides a proof-of-concept for the integration of radiological and immune-inflammatory metrics and a solid foundation for future research in this patient population. Next, we plan to expand the sample size, enrich sample diversity, incorporate more direct immunological variables and optimize model parameters to make model predictions more accurate.

To further validate and improve the generalizability of our model, future multi-institutional external validation studies are planned. Collaboration with other centers will enable assessment of the model’s performance across diverse clinical settings and patient populations.

This study has several limitations inherent to its design. First, the retrospective, single-center nature may introduce selection bias and limits the generalizability of our findings, a concern compounded by the lack of external validation. Second, the absence of detailed data on systemic therapies administered before or after SRS represents a key unmeasured confounder for overall survival. Third, our analysis lacked comprehensive molecular profiling and detailed data on the tumor immune microenvironment, which restricts the biological depth and contemporary relevance of our cohort. Finally, the prognostic value of systemic inflammation was assessed only by a single baseline NLR measurement, failing to capture its potentially more informative dynamic changes during treatment. Future studies should investigate the role of serial NLR measurements in refining prognostic models.

## Conclusion

In summary, immune-inflammatory (NLR) and radiological-clinical (volume of BM, SIR, EI, maximum diameter, age) factors were independent predictors for the efficacy of SRS therapy and OS in NSCLC patients with BM. Novel user-friendly decision tree and nomogram models were developed, which incorporate the systemic immune-inflammation profile, to assist clinicians in optimizing clinical management for this patient population.

## Data Availability

The raw data supporting the conclusions of this article will be made available by the authors, without undue reservation.
