# Supplementary material for: Immuno-inflammatory signature for predicting therapeutic response and survival after stereotactic radiosurgery in NSCLC patients with brain metastases: a retrospective cohort study
Source: Front Immunol. 2025 Dec 18;16:1739812. doi: 10.3389/fimmu.2025.1739812 (PMC12756399; doi:10.3389/fimmu.2025.1739812)
Supplement: Supplementary file 1 [file Table1.docx]

| **Supplementary Table S1. Treatment Response at 3 Months According to RANO-BM Criteria** | | | |
| --- | --- | --- | --- |
|  |  |  |  |
|  |  |  |  |
| Response Category | Whole cohort (n=464) | Training sample (n=325) | Validation sample (n=139) |
| Complete Response (CR) | 98 (21.1%) | 70 (21.5%) | 28 (20.1%) |
| Partial Response (PR) | 218 (47.0%) | 150 (46.2%) | 68 (48.9%) |
| Objective Response Rate (ORR) | 316 (68.1%) | 220 (67.7%) | 96 (69.1%) |
| Stable Disease (SD) | 99 (21.3%) | 70 (21.5%) | 29 (20.9%) |
| Disease Control Rate (DCR) | 415 (89.4%) | 290 (89.2%) | 125 (90.0%) |
| Progressive Disease (PD) | 49 (10.6%) | 35 (10.8%) | 14 (10.0%) |

| **Supplementary Table S2**. **Performance (C-index) of the Nomogram in Key Clinical Subgroups of the Validation Set** | | |
| --- | --- | --- |
|  |  |  |
|  |  |  |
| Subgroup | n | C-index (95% CI) |
| Overall Validation Set | 139 | 0.66 (0.53 - 0.80) |
| EGFR Mutation Status | | |
| Mutation | 70 | 0.65 (0.48 - 0.82) |
| Wild-type | 69 | 0.67 (0.49 - 0.85) |
| Extracranial Metastasis | | |
| Yes | 69 | 0.64 (0.45 - 0.83) |
| No | 70 | 0.68 (0.51 - 0.85) |
| Age Group | | |
| ≤ 48 years | 18 | 0.69 (0.41 - 0.97)* |
| > 48 years | 121 | 0.65 (0.52 - 0.79) |
| Number of Brain Metastases | | |
| 1 | 72 | 0.67 (0.51 - 0.83) |
| 2-4 | 67 | 0.65 (0.46 - 0.84) |

| **Supplementary Table S3. Sensitivity Analysis: Multivariable Cox Regression for Overall Survival Using Original Continuous Variables** | | |
| --- | --- | --- |
|  |  |  |
|  |  |  |
| Variable | HR (95% CI) | *P* Value |
| Age (per 1-year increase) | 1.04 (1.02 - 1.07) | < 0.001 |
|  |  |  |
| Edema Index (per 1-unit increase) | 1.22 (1.11 - 1.34) | < 0.001 |
|  |  |  |
| Volume of Brain Metastasis (per 100 cm³ increase) | 1.08 (1.05 - 1.11) | < 0.001 |
|  |  |  |
| Age (per 1-year increase) | 1.04 (1.02 - 1.07) | < 0.001 |
|  |  |  |
| Score Index for Radiosurgery (SIR) | | |
|  |  |  |
| Grade I (Reference) | 1 | < 0.001 |
|  |  |  |
| Grade II vs. I | 0.40 (0.24 - 0.68) | 0.001 |
|  |  |  |
| Grade III vs. I | 0.21 (0.11 - 0.40) | < 0.001 |


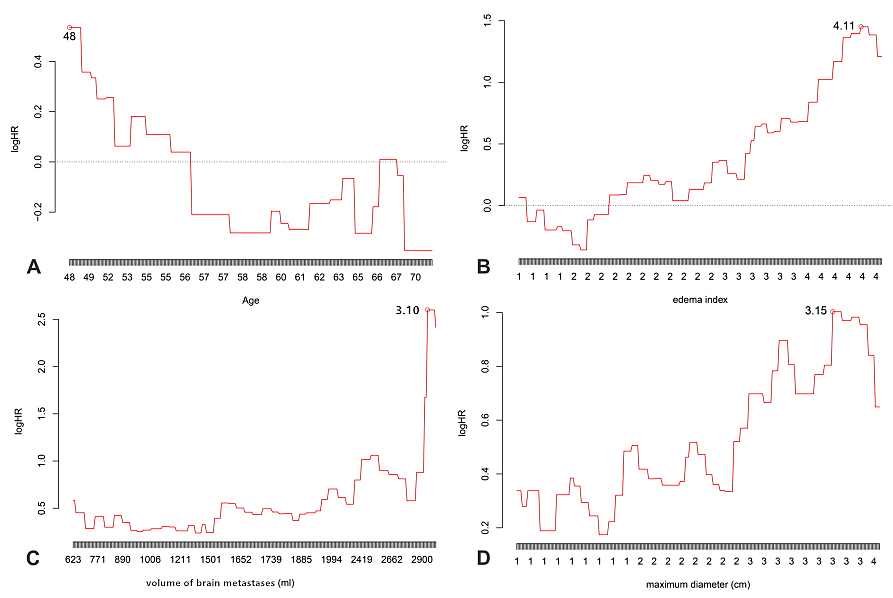


**Figure S1**. The results of running Log-rank tests, which indicated that the optimal cut-off points for age (A), edema index (B), volume of brain metastasis (C) and maximum diameter (D) were 48, 4.11, 3.15 cm and 3.10 cm^3^, respectively.


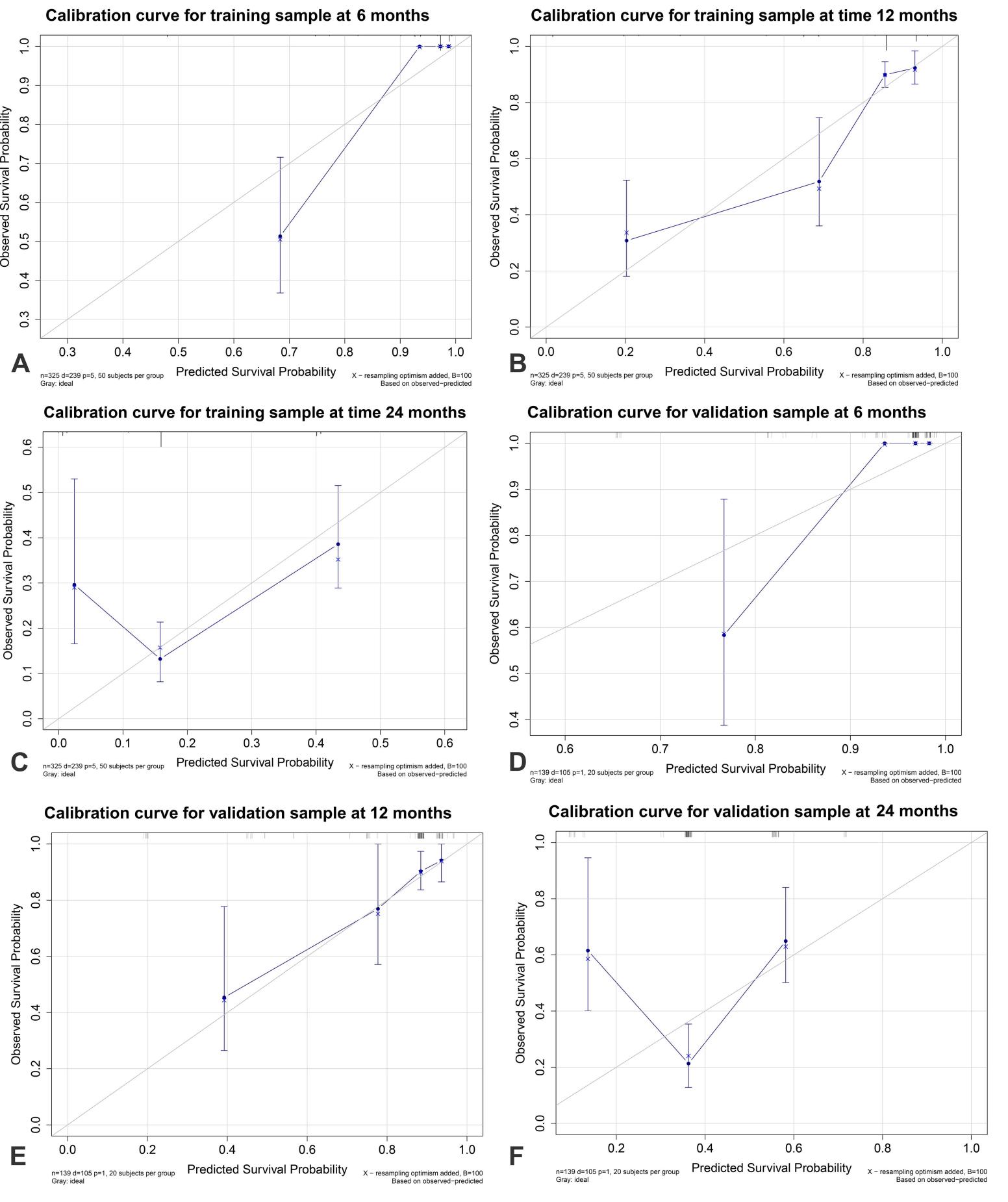


**Figure S2**. The calibration curves at 6, 12, and 24 months, for training (A-C) and validation (D-F) samples. Favourable consistency between predicted and the actual survival rates are demonstrated.
